# Promotion of Parenting and Mental Health Needs among Chinese Women Living in Japan: A Qualitative Study

**DOI:** 10.3390/ijerph192013538

**Published:** 2022-10-19

**Authors:** Yunjie Luo, Yoko Sato, Tianyue Zhai, Hiromi Kagamiyama, Yasuhiko Ebina

**Affiliations:** 1 Graduate School of Health Sciences, Hokkaido University, Sapporo 0600812, Japan; 2 Faculty of Human Sciences, Hokkaido Bunkyo University, Eniwa 0611449, Japan; 3 Department of Obstetrics and Gynecology, School of Medicine, Hokkaido University, Sapporo 0608638, Japan; 4 Department of Nursing Faculty of Health Sciences, Japan Healthcare University, Sapporo 0620053, Japan; 5 Faculty of Health Sciences, Hokkaido University, Sapporo 0600812, Japan

**Keywords:** mental health, emigrants and immigrants, women, parenting, social support, qualitative study, thematic analysis

## Abstract

Chinese women raising children in Japan tend to experience high parenting stress and poor mental well-being. However, their specific parenting and mental health promotion needs remain unknown. This study aimed to explore the parenting and mental health promotion needs of Chinese women living in Japan and provide recommendations to guide interventions. Semi-structured in-depth interviews were conducted. Participants included 15 women aged 28–39 years who were pregnant or rearing a child younger than six years old. Thematic analysis was performed for data analysis. More than half of the participants experienced mental health problems, such as depressive symptoms and child-rearing stress. Four themes relating to their needs were identified: concrete support, information provision, caring and understanding, and social network building. Information provision and social network building should be emphasized as practical social support mechanisms to improve these women’s mental health. Furthermore, a mental health promotion intervention should be developed to address this vulnerable population’s needs. Healthcare providers and public health workers should help improve the social support systems of Chinese women in Japan to prevent mental health problems. Potential transcultural education can, arguably, help healthcare providers better understand transcultural care.

## 1. Introduction

The number of international migrants was approximately 281 million in 2020, accounting for 3.6% of the global population [1]. The health determinants of international migrants vary considerably between the countries of migration and destination due to their different backgrounds [2]. Meanwhile, the process of migration and resettlement is recognized as a stress-inducing phenomenon associated with high mental health risks [3]. Thus, international migration and mental health is a highly complex and nuanced topic that must be explored—particularly, immigrant women’s mental health. 

Women exhibit a high risk of postpartum depression during pregnancy and post childbirth [4]. Compared with the native population, the immigrant population has been identified as experiencing greater problems in depression and anxiety [5]. Thus, immigrant women might exhibit exceptionally higher risk than non-immigrant women. A systematic review reported that immigrant women from low- and middle-income countries experienced perinatal depression relating to their children’s mental health and highlighted social support as a protective factor [6]. Consequently, providing mental health support for immigrant women is recommended to prevent postpartum depression and strengthen public health policy [7].

As of December 2021, the number of foreigners living in Japan was 2,760,635 [8]. The high proportion of foreigners in Japan has resulted in the psychological well-being of the immigrant population becoming a significant public health issue [9]. In Japan, as immigrant populations are not a homogeneous group, their health issues should be considered and studied separately per their various backgrounds. Chinese nationals constitute the largest group in Japan, with 716,606 Chinese nationals accounting for 26.7% of the foreign population [8]. For status of residence among Chinese residents in Japan, “permanent resident” status accounted for the majority at 41.4%, followed by “student” status at 13.5%, and “technical, humanities, and international services” status at 11.3% [8]. Since Japan is not a traditional immigrant country, as a general rule, applicants became eligible to apply for permanent residency after 10 continuous years of residency in Japan. Thus, the current major reasons for Chinese residents living in Japan are study and work. Chinese women are the most numerous foreign female population in Japan, accounting for 27.8% of the total [8]. A literature review found a high prevalence of postpartum depression in Chinese immigrant women [10]. A previous study indicated that immigrant Chinese, new mothers in Japan are likely to exhibit a high risk of postpartum depression, general stress, and cross-cultural stress [11]. Moreover, Chinese women living in Japan with preschool-aged children reported high parenting-related stress and expressed a desire for childcare support [12]. Furthermore, a survey on the general population of immigrant Chinese women in Japan indicated that high coronavirus disease 2019 (COVID-19) concerns were related to poor mental health [13]. Therefore, improving this vulnerable population’s mental health—especially during the emergency pandemic period—is necessary.

Few studies have been conducted on improving the mental health of Chinese immigrant women in Japan. A systematic review including 36 studies reported that immigrant perinatal women in Japan experienced varied physical, psychological, social, and economic problems and recommended social support to resolve them [14]. However, only one study in this review included multiracial women such as Chinese women [15]. Additionally, some surveys that were administered in Japan for Chinese mothers with children of varying ages revealed that Chinese mothers exhibited parenting stress and loneliness, and lacked social support [16,17,18]. Furthermore, Jin et al. developed a nursing intervention to prevent postpartum depression among Chinese immigrant women in Japan, but no significant results were reported [19]. Therefore, the specific needs of these women regarding parenting and mental health promotion should be identified to provide effective social support for this specific population and improve their mental health. This study addressed the research question, ‘What are the parenting and mental health needs of Chinese women living in Japan?’.

Qualitative research is a useful methodology that helps health and nursing science researchers understand the concerns of participants, guide emerging theories, and describe social support’s health impact [20]. Thus, qualitative study design was considered the ideal methodology to explore our aims. This study aimed to explore parenting and mental health promotion needs to provide development recommendations for an intervention promoting the mental health of Chinese women in Japan.

## 2. Materials and Methods

A qualitative descriptive research design was employed to demonstrate the needs of Chinese immigrant women in Japan regarding their experience of parenting and mental health [21,22]. This study conducted semi-structured interviews to guide participants on pertinent topics while providing the necessary flexibility to investigate new data [23].

### 2.1. Participants and Recruitment

This study used the convenience sampling technique. Participants were recruited from various online groups using WeChat, a Chinese multi-purpose messaging social media platform developed by Tencent. Online groups of Chinese people living in Japan include consumer groups, second-hand goods exchange groups, and associations of Chinese students. Thereafter, snowball sampling was used to recruit more participants. The recruitment of study participants was stopped after attaining data saturation. Approximately 90% of codes emerged after 12 interviews in a qualitative study [24].

Women who met the inclusion criteria were recruited. The inclusion criteria were as follows: (1) Chinese women aged over 20 years; (2) experience of pregnancy or of having reared at least one child under the age of six years in Japan; (3) willingness to be interviewed; (4) ability to speak, read, and understand Mandarin Chinese; and (5) no diagnosed mental disorders or intellectual disabilities.

### 2.2. Data Collection

The interview guide (see Table 1) was designed following a literature review and a previous study that indicated the mediating effect of parenting stress between social support and mental health among Chinese women living in Japan—based on the health effect of social support methodology [16,25,26]. Thus, data collection included questions related to (1) demographic factors; (2) pregnancy, childbirth, and the child rearing experience; (3) mental health; and (4) an extended, open-ended question to explore more related experiences that the participants would like to narrate. 

Before conducting the interviews, all participants read the informed consent form, expressed their willingness to be interviewed, and provided the required information. Participants could quit the interviews at any time without providing a reason for the same, and all relevant data would be withdrawn. Per the participants’ convenience, interviews were conducted either face-to-face or online via Zoom. Face-to-face interviews were conducted in private areas, such as the interview room of the affiliated institution or the participant’s home. Interviews were conducted in Mandarin Chinese from October to November 2021. Data were collected by the first author, who is a Chinese woman living in Japan, a native speaker of Mandarin, and a nursing researcher with experience in conducting child-rearing salons, focus group discussions, and in-depth interviews among foreign women living in Japan. Interviews lasted 25–60 min and were audio-recorded with the participants’ permission. After the interviews, each participant received a gift card valued at 20 USD. To protect the participants’ privacy, all collected data were coded with numbers (such as ID1, ID2, etc.) and stored using a password after personal information had been removed.

### 2.3. Data Analysis

The collected data were analysed using NVivo 12.0 software. Data were analysed using the thematic analysis method [27]. To guarantee the trustworthiness of this study, credibility, transferability, dependability, and confirmability were considered [28]. First, each recording was transcribed, read, and reread in Mandarin Chinese by a native speaker. Second, initial codes were created and reviewed. Eighty-one initial codes were generated. The next level of analysis included reviewing the codes and translating them into English. The process of code generation and translation were independently conducted by two Chinese-English bilingual researchers in the field of medical (T.Z.) or nursing sciences (Y.L.). If any differences or disagreements arose, a third researcher (Y.E.) assisted in deciding. The translated data were further checked by an English native speaker. In this step, the variety of the initial codes was carefully kept to generate higher-level sub-themes. Initial codes with the same essence were categorised and refined into sub-themes and themes. The coding technique and process of generated themes and sub-themes were reviewed, discussed, and named by all the research team members. 

## 3. Results

Seventeen women were approached; two were excluded as they did not meet the inclusion criteria and did not complete the interviews. Finally, 15 participants were interviewed and analysed. Among the 15 participants, 10 were recruited from various online groups, and 5 were recruited using the snowball sampling method.

### 3.1. Participants’ Demographic Factors

Table 2 presents the participants’ demographic factors. Participants’ mean age was 32.7 (SD = 3.0), ranging from 28 to 39 years. The mean length of residence duration was 7.8 (SD = 4.4) years. Roughly half (8) of the participants had two children, 9 were employed, 8 had a middle level of annual household income, and 8 had a graduate school educational background. Further, 6 had non-work permit residence status, 13 lived in a nuclear family structure, 9 had a middle or low level of Japanese proficiency, and 14 were married to a Chinese man.

### 3.2. Participants’ Parenting and Mental Health Promotion Needs

We identified four themes regarding the participants’ parenting and mental health needs: concrete support, information provision, caring and understanding, and social network building. Table 3 presents the 4 themes and 15 sub-themes.

#### 3.2.1. Concrete Support

Participants reported both negative and positive experiences during their pregnancy, childbirth, and child rearing. All the participants who had positive parenting and mental health experiences obtained sufficient instrumental support. Providing sufficient concrete support for Chinese women living in Japan would effectively prevent or alleviate the effects of negative parenting and mental health experiences. We addressed the concrete support that the participants desired, including perinatal care support, childcare support, and language support. 

Despite being in Japan, more than two-thirds of the participants practised ‘Zuoyuezi’. ‘Zuoyuezi’ is a traditional Chinese month-long postpartum care ritual, which refers to the postpartum ‘sitting month’ period in Chinese. It encompasses several aspects, including house confinement for a month, support from husbands, parents, or in-laws, and strict rules for perinatal behaviours such as specific diets and no hair washing. To help them during the postpartum period, their parents from China often visited them in Japan to provide support.

*ID 7: I received the care of ‘Zuoyuezi’ after giving birth… The tradition was performed based on my parents’ experience.*

*ID 10: After I gave birth to my first child, there was no COVID-19, and my mum came to Japan to help me perform ‘Zuoyuezi’.*

However, the COVID-19 outbreak prevented Chinese women living in Japan from obtaining support from their families in China. Some participants, therefore, relied on support from their friends or used a postnatal care service.

*ID 2: My family could not come to Japan because of COVID-19 … At first, I wanted to return to China, but the long quarantine period and expensive cost made me give up… I was anxious. Because my family is more experienced in taking care of postnatal care… Finally, I used a postnatal care service, but I could only use it for one week.*

*ID 10: There were two Chinese pregnant women in my community when I had my second child. Our delivery dates were close, so we helped each other after giving birth to help the care of Zuoyuezi… Because of COVID-19, none of our family members could come to Japan.*

Some participants experienced parenting-related stress because of the difficulty in balancing child rearing and work, study, or daily life. Childcare and housework support from their family allowed them private time to balance child rearing and daily life.

*ID 9: Sometimes, I do not have time to eat lunch because I have to look after my kid… It would be nice to have someone to help me, so I can have time to go to the toilet or take a bath… Taking care of a child without help does not leave me with enough time.*

*ID 3: It is best to have a family member come over to Japan to help with cooking or share the housework. Now that I live with my mother-in-law, she helps me a lot, and I feel relaxed.*

Participants with a low level of Japanese proficiency usually needed language support from their husbands or friends. The Chinese medical interpretation group was mentioned as particularly helpful during pregnancy, birth, and child rearing.

*ID 9: I came to Japan with no Japanese language skills, so the [main] difficulty in the whole process of childbirth and child rearing was the language barrier.*

*ID 7: The difficulty is that every time I go for a maternity check-up, I have to ask my husband to take time off work to accompany me because I do not speak Japanese.*

*ID 1: I think it is important that I contact the Chinese medical interpretation group. They were helpful. They helped me to go to the health centre to get a mother and child handbook, apply for subsidies, and to make appointments for maternity check-ups. They continued to help me for the next two years.*

One participant experienced difficulty renting a house because she was a pregnant foreign woman. Thus, essential life support should be provided for this population to improve their daily life in Japan.

*ID 1: A stressor for me during my pregnancy was renting a house. I lived in the university’s dormitory. I was told I could not live in the dormitory if I had a baby. I was anxious and stressed. I had a terrible experience finding a place to live… Because I was not good at Japanese and was a pregnant foreigner, there was no suitable place to live… Many landlords did not want to rent to pregnant foreign women like me. I did not feel very good at the time.*

#### 3.2.2. Information Provision

Almost all the participants experienced a difficult period during which they lacked sufficient information, especially when they were newcomers in Japan, had a low level of Japanese proficiency, or had no local family and friends. Most participants mentioned that they lacked child-rearing knowledge and wanted to obtain related information conveniently, such as information on vaccinations, weaning, and caring for a sick baby.

*ID 6: I am interested in how to make myself better at child rearing. Knowledge of child rearing is what I need most because that will affect my mood… The first is about baby vaccinations. I feel my baby needs a lot of vaccinations, and my baby will probably be back in China in a few months. I would like to know how the baby vaccination systems differ in China and Japan. The other is about the baby’s food, such as how to wean her from breast milk or balance breast milk and complementary food. I would also like to know what to do if my baby is unwell, such as experiencing flatulence, hiccups, fever, and vomiting. If it is severe, which hospital should I go to? These are the things I am worried about.*

*ID 10: When I had my first child, I did not have many friends, so I had fewer sources of information.*

*ID 11: I faced different concerns at different ages. It would be nice to have an online communication website, which includes various sections with diverse knowledge. Mothers like me can get the information conveniently.*

Some participants indicated that they had trouble because of the differences between the Chinese and Japanese medical and educational systems or because they did not know about Japan’s welfare policy for pregnant women. Therefore, necessary information to help foreign women become familiar with Japanese medical and educational policies should be provided.

*ID 5: If there was one thing that made me feel a little uncomfortable, it would be that the test terms of maternity check-ups in Japan are different from those in China… I found that in Japan, they do not perform Down’s syndrome screening tests, even in women aged over 35 years. The doctor would not even talk to us about it… My husband and I asked the doctor to screen for Down’s syndrome, even if we had to pay for it ourselves.*

*ID 14: It was difficult when my child entered kindergarten because there was no one to ask about the preparations and admission procedure.*

*ID 4: I became pregnant after leaving my last job. Because I had worked at my new company for less than a year and received an unemployment subsidy at my previous company, I could not receive a childcare subsidy now… If I could get my childcare subsidy back, I would be willing to pay back double the unemployment subsidy.*

Some participants mentioned that if they had known some of the information earlier, they might have had an easier time raising their children in Japan. Thus, general information support is necessary, especially for newcomers.

*ID 1: After living in Japan for a long time, I knew that there were some online groups for Chinese speakers in Japan where one could buy second-hand furniture or find people to move house. If I had known this earlier, it would have been helpful*.

#### 3.2.3. Caring and Understanding

Almost all the participants mentioned that they experienced a difficult emotional period after giving birth and during child rearing. More than half the participants had severe mental health problems during postpartum and child rearing, including depressive symptoms, loneliness, anxiety, and parenting stress. Caring and understanding from their husband were crucial, especially for first-time mothers. Insufficient support and a lack of comprehension of mental health issues from their husbands considerably affected the participants’ psychological well-being.

*ID 8: I had postnatal depressive symptoms… I would look at my children and want to cry… Because of the family conflict between my mother-in-law and me, my husband supported his mother unconditionally… I raised our children alone; my husband did not help much, and my mother-in-law did not help either… I was depressed and even suicidal when I had my first child.*

*ID 13: When I had my first child, I had severe depressive symptoms. I thought about bad things and could not perceive the value of life… It was because of pressure, partly from my mother and partly from my husband, that he was not good enough.*

Some participants actively communicated with people around them because communication alleviated their negative emotions, such as loneliness. The main individuals for the participants to communicate with were usually their mothers and other Chinese mothers living in Japan.

*ID 4: Luckily, I have a few Chinese mothers around me who can communicate and help me to relieve my stress because we can spill the beans to each other.*

*ID 12: I felt very lonely when I had my first child because my husband had to work. I always take my kid to the park and talk to other mums… In winter, I would take my kid to the supermarket or store because I wanted to find someone to talk to me.*

Most participants stated that to cope with negative emotions, they used different methods, such as shopping, doing housework, eating sweets, reading books, spending money, earning money, and exercising. Additionally, some of them desired to obtain advice from a mental health specialist.

*ID 12: I feel like what I need is psychological guidance. When I faced mental health problems … I needed someone to help me solve them or unblock them.*

#### 3.2.4. Social Network Building

Several participants took care of their children by themselves, which led them to experience extreme social isolation. Therefore, helping Chinese women build a social network in Japan is vital to overcome social isolation.

*ID 11: From a psychologically perspective, for example, if I encounter any problems or something happens, there is no one around to talk to, except my husband. My relatives, parents, and close girlfriends are not in Japan, which may lead to my psychological anxiety*.

More than half of the participants wished for someone with whom they could share their parenting experiences and build a long-term connection, especially other mothers who were raising same-aged children. Helping Chinese women build a social network, such as by organising a child-rearing communication group, would help them attain greater parenting experience and adapt to life in Japan.

*ID 6: I think, for example, a seminar … we can have a chance to talk to other mothers, to make friends, have a place to talk, and share [our thoughts] with each other… Because sometimes, for pregnant women, it is difficult to move around and communicate with the outside world, but they still need to talk or communicate… If possible, it is good to have this kind of organisation.*

*ID 11: I hope there was a group around me, for example, using LINE or WeChat, where I could easily ask at any time, and then someone would share her experience. That would be better.*

Some participants mentioned that they wanted to maintain a strong connection with the community from which they could conveniently receive childcare support and desired greater attention from the authorities.

*ID 15: I would like to see more child-rearing seminars in my community, so that I can always ask questions when my children are growing up.*

*ID 4: I hope the ward office will hold events for childcare mothers. I am not quite sure if there was one before COVID-19… I also hope that the Japanese government will pay more attention to us foreigners; [it is] not that I want more benefits, but I hope there will be more attention.*

## 4. Discussion

This study explored the parenting and mental health promotion needs of Chinese women living in Japan to provide evidence supporting their child-rearing experience. It highlights the importance of social support, especially their husbands’, in preventing mental health problems and alleviating stress. Moreover, our findings suggest that an effective intervention on mental health promotion should be developed for Chinese women living in Japan. Social support from the neighbourhood or community should be provided to immigrant and resettled women during periods such as the COVID-19 pandemic. 

Potential overlapping exists in some quotes pertaining to the themes, such as a quote expressed building a social network simultaneously to obtain necessary information. The function of quotes is to provide evidence for the sub-themes or themes, illustrate a concrete example, and present participants’ thoughts and feelings [29]. It is common and acceptable in qualitative research for a quote to fit into multiple themes, indicating a connection between them [27]. This intrinsic connection could inspire health providers and policymakers when it comes to providing support or making decisions.

The participants in this study reported considerable mental health problems during pregnancy, childbirth, and child rearing, such as postpartum depression, anxiety, parenting stress, and loneliness. These mental health problems were also previously found among Chinese women living in Japan [16,17,18]. We found that receiving support from their husband was the most important factor related to women’s perinatal mental health. Childcare support from family members was another essential factor in preventing or reducing Chinese women’s child-rearing stress and postpartum depression. In China, mothers and mothers-in-law are important providers of child rearing and postpartum care support. They also generally share the responsibility of rearing grandchildren to reduce their children’s parenting burden [30]. This concurs with this study’s findings. However, the relationship between women and their family members, especially their mothers-in-law, plays an essential role in relation to psychological well-being [31]. 

‘Zuoyuezi’ is a postpartum care tradition that is highly valued by Chinese women to recover their physical and mental health and prevent future diseases [32]. Most participants in this study adhered to this tradition. To practice ‘Zuoyuezi’, obtaining the support of an experienced person, such as a mother or mother-in-law, is necessary. However, the COVID-19 outbreak made it difficult to obtain family support [13]. Thus, providing postpartum care for Chinese women living in Japan is essential to help them get through the most challenging postpartum period, such as by extending the available period of postpartum care services. 

In 2006, the Japanese Ministry of Internal Affairs and Communication developed a Multicultural Coexistence Promotion Plan to help foreign residents adapt to life in Japan [33]. However, 49% of the local governments had not implemented the plan as of April 2022 [34]. Meanwhile, previous studies have reported that despite having been offered support, foreign women in Japan continue to experience difficulties associated with child-rearing and acculturation [14,35]. Therefore, healthcare providers should consider a practical approach to increase the utilisation of health care services among immigrant women, such as examining mental health treatment differences by country of origin [36]. A possible transcultural education of healthcare providers can also be considered to help providers understand postpartum traditions and facilitate women in following their customs. For example, ‘Zuoyuezi’ can be aided via offering home visits to help women who choose to spend their first postpartum month at home. Home visits might be an effective intervention approach to improve immigrant women’s mental health [37]. Furthermore, a more understanding and knowledgeable crew of healthcare professionals might help the mother feel more connected to them and the healthcare system in Japan.

Immigrant and resettled women rearing children in a new environment not only experience stress from the process of childcare but also face challenges in building new lives [38]. Immigrants in a strange environment lack essential information resources; obtaining reliable health information and accessing health services becomes a significant social issue [39]. Therefore, information barriers should be addressed practically to help them adapt to general life and child rearing in Japan. Making information convenient to obtain and easy to understand is essential. A previous study reported that low Japanese proficiency limited the communication of immigrant women in Japan with health providers, thereby reinforcing their loneliness [40]. Based on the study participants’ responses, we recommend providing online information and material in immigrant women’s native language in future studies. This will ensure that perinatal health-related information is linguistically accessible and culturally inclusive [41].

Making friends, especially with women from the same cultural background, is a practical way for immigrant women to adapt to life in a different environment by sharing their experiences and enhancing their sense of belonging. For immigrant women, a sense of belonging might decrease their loneliness and homesickness and protect their mental health [42]. Thus, helping immigrant women build a social network within their community is necessary. Establishing close connections will assist immigrant women to access community services and resources [43]. Community ties have played an essential role in assisting immigrant women, especially during the COVID-19 pandemic. These findings imply that social support providers, healthcare providers, and public health workers should help immigrant and resettlement women strengthen their connection with the community and neighbourhood to ensure that they do not lack social support during challenging periods such as the current COVID-19 pandemic. Internet-based intervention played a significant role in preventing mental health issues in various populations during COVID-19 [44,45]. Thus, we recommend developing an Internet-based intervention to help Chinese women in Japan obtain information conveniently and efficiently. We also recommend provide an available approach to assist them in building a community tie even during an extraordinary period, such as online child-rearing salons. This qualitative study has several limitations that must be considered. First, the sample size was small, which may affect the generalizability of the results. Additionally, the participants were recruited using convenience and snowballing sampling, and primary recruitment was only conducted using WeChat, which might have introduced technological bias. Moreover, due to COVID-19, we could not interview all the participants in person. The different interview methods might have influenced the quality of the data collected. Finally, the qualitative study design limits the generalizability of the findings. However, the participants’ experiences and considerations were explored in depth.

## 5. Conclusions

This study explored the needs of Chinese women living in Japan concerning parenting and mental health problems to provide evidence for developing practical interventions to improve their mental well-being. More than half of the participants experienced mental health problems during postpartum and child rearing, such as depressive symptoms and parenting stress. Four themes regarding their needs were identified: concrete support, information provision, caring and understanding, and social network building. Information provision and social network building should be emphasized as practical social support mechanisms to improve the mental health of Chinese women living in Japan. Support from the neighbourhood and community is essential for participants, particularly during the COVID-19 pandemic. Based on these findings, an effective mental health promotion intervention should be developed for Chinese women living in Japan. Additionally, a possible transcultural education could be considered to help healthcare providers understand transcultural care when they interact with immigrants.

## Figures and Tables

**Table 1:** Interview guide card.

Topics	Prompts
Demographic factors	Age, number of children, duration of residence, employment status, annual household income, education background, residential status, family structure, Japanese proficiency, nationality of spouse
Pregnancy, childbirth, and child-rearing experience	Please tell me about your experience of pregnancy, childbirth, and child rearing in Japan.
	What kind of difficulties did you experience during pregnancy, childbirth, and child rearing in Japan?
	What caused these difficulties?
	How did you overcome these difficulties?
	What kind of support did you receive or wished to receive when going through these difficulties?
Mental health experience	What do you think about the term ‘mental health problems’?
	Did you experience any mental health problems during pregnancy, childbirth, and child rearing in Japan?
	What caused these mental health problems?
	How did you address these mental health problems?
	What kind of support did you receive or wished to receive when experiencing these mental health problems?
An extended question	If you have any other experiences that you would like to share, please feel free to do so.

**Table 2:** Participants’ demographic factors (*n* = 15).

Demographic Factors	Number (%)
Age (years)	
28–34	10 (66.7%)
35–39	5 (33.3%)
Number of children	
1	7 (46.7%)
2	8 (53.3%)
Duration of residence (years)	
<5	3 (20%)
5–10	8 (53.3%)
>10	4 (26.7%)
Employment status	
Employed	9 (60%)
Unemployed	6 (40%)
Annual household income (USD)	
<30,000	4 (26.7%)
30,000–70,000	8 (53.3%)
>70,000	3 (20%)
Educational background	
Junior college	2 (13.3%)
University	5 (33.3%)
Graduate school	8 (53.3%)
Residence status	
Work permit ^1^	5 (33.3%)
Non-work permit ^2^	6 (40%)
Family permit ^3^	4 (26.7%)
Family structure	
Nuclear family	13 (86.7%)
Extended family	2 (13.3%)
Japanese proficiency	
High	6 (40%)
Middle	4 (26.7)
Low	5 (33.3%)
Nationality of spouse	
Chinese	14 (93.3%)
Japanese	1 (6.7%)

^1^ Work permit includes medical services, engineer/specialist in humanities/international services, and highly skilled professional; ^2^ Non-work permit includes student and family stays; ^3^ Family permit includes permanent resident and spouse of permanent resident.

**Table 3:** Parenting and mental health promotion needs. (*n* = 15).

Theme	Sub-Theme (*n*, %)
Concrete support	Perinatal care support (15, 100%)
	Language support (8, 53.3%)
	Childcare support (15, 100%)
	Housework support (5, 33.3%)
	Everyday living support (4, 26.7%)
Information provision	Child rearing information and knowledge (11, 73.3%)
	Familiarity with Japanese medication, education, and policy (8, 53.3%)
	General life information (4, 26.7%)
Caring and understanding	Husband’s support (14, 93.3%)
	Communicate with someone (10, 66.7%)
	Various methods to cope with negative emotions (73.3%)
	Advice from mental health specialists (3, 20%)
Social networking building	Overcome social isolation (4, 26.7%)
	Child rearing communication group (10, 66.7%),
	Strong community ties (9, 60.0%)

## Data Availability

The transcribed data that support the findings of this study are available on request from the corresponding author. The primary data are not publicly available due to protect the privacy of participants.
